# Supplementary material for: Shortcut to chemically accurate quantum computing via density-based basis-set correction
Source: Commun Chem. 2024 Nov 18;7:269. doi: 10.1038/s42004-024-01348-3 (PMC11574143; doi:10.1038/s42004-024-01348-3)
Supplement: Supplementary file 2 — Supplementary Material [file 42004_2024_1348_MOESM2_ESM.pdf]

Supplementary Information: Shortcut to chemically accurate quantum computing via density-based basis-set correction

Diata Traore,[1,2] Olivier Adjoua,[1] Cesar Feniou,[2,1] Ioanna-Maria Lygatsika,[3,1,*] Yvon Maday,[3,4] Evgeny Posenitskiy,[2] Kerstin Hammernik,[5] Alberto Peruzzo,[2] Julien Toulouse,[1,4] Emmanuel Giner,[1] and Jean-Philip Piquemal[1,2,†]

[1] *Sorbonne Université, LCT, UMR 7616 CNRS, 75005 Paris, France*
[2] *Qubit Pharmaceuticals, Advanced Research Department, 75014 Paris, France*
[3] *Sorbonne Université, LJLL, UMR 7598 CNRS, 75005 Paris, France*
[4] *Institut Universitaire de France, 75005 Paris, France*
[5] *NVIDIA Corporation, Santa Clara, CA, USA*

CONTENTS

I. Mapping Quantum Chemistry to Quantum Computers and Quantum Solver 1

II. Data 3
A. Geometries 3
B. Total ground-state energies with standard basis sets 3
C. Total ground-state energies with SABS 6
D. Dissociation curves 14
E. Dipole moments 17

III. SABS 18
A. H_2, SABS/V5Z-8 18
B. LiH, SABS/V5Z-4 19
C. LiH, SABS/V5Z-7 20
D. LiH, SABS/V5Z-10 21
E. H_2O, SABS/V5Z-10 22
F. N_2, SABS/V5Z-6 23

IV. ADAPT-VQE energy convergence 24

References 32

I. MAPPING QUANTUM CHEMISTRY TO QUANTUM COMPUTERS AND QUANTUM SOLVER

In a given spin-orbital basis set $\mathcal{B}$, the molecular electronic Hamiltonian can be expressed in second-quantization as

$$\hat{H} = \sum_{p,q} h_{pq} \hat{a}_p^\dagger \hat{a}_q + \sum_{p,q,r,s} w_{pqrs} \hat{a}_p^\dagger \hat{a}_q^\dagger \hat{a}_s \hat{a}_r, \tag{1}$$

where the indices p, q, r, s run over the spin-orbitals, $\hat{a}_p$ and $\hat{a}_p^\dagger$ are the pth fermionic annihilation and creation operators, and h_{pq} and w_{pqrs} are the one-electron and two-electron integrals. The Jordan-Wigner and Bravyi-Kitaev transforms [1, 2] are usually employed to map annihilation and creation operators to tensor products involving Pauli matrices, enabling the representation of the second-quantized Hamiltonian $\hat{H}$ on a quantum computer, with each qubit encoding a spin-orbital of the system. The molecular Hamiltonian $\hat{H}$ now expressed as linear combination of Pauli products, various quantum algorithms such as the Variational Quantum Eigensolver (VQE) [3] and Quantum Phase Estimation (QPE) [4, 5] can compute the ground state of this molecular Hamiltonian [6–8]. In this paper,

* Present address: CEA, DAM, DIF, F-91297 Arpajon, France; and Université Paris-Saclay, LMCE, 91680 Bruyères-le-Châtel, France
† jean-philip.piquemal@sorbonne-universite.fr

a VQE-inspired algorithm is used. The VQE minimizes the Hamiltonian's expectation value with respect to a parameterized ansatz wave function, in a classical-quantum hybrid approach. The challenge is constructing an ansatz wave function balancing accuracy with a shallow quantum-circuit representation for NISQ devices. To address such a challenge, the adaptive derivative-assembled pseudo-Trotter variational quantum eigensolver (ADAPT-VQE) [9] has emerged as a standard, by proposing an ansatz that is dynamically grown through an iterative process, resulting in an increased accuracy with shallower circuits than traditional VQE ansatze, for which the structure is predetermined before simulations. The general workflow of the ADAPT-VQE algorithm is as explained in Ref. [10].

In the present study, we use the Qubit-Excitation-Based pool of operators, which is considered a standard [11]. Note that one issue of ADAPT-VQE is linked to its classical optimization procedure that can encounter barren plateaus generating very large numbers of parameters and therefore limiting practical convergence. In such cases, a variant of ADAPT-VQE can be used: the Overlap-ADAPT-VQE [12] that allows one to grow wave functions by maximizing their overlap with an intermediate target wave function. That way, when barren plateaus are encountered, Overlap-ADAPT-VQE can reduce the number of optimization parameters and produce an ultra-compact ansatz suitable for high-accuracy initialization of a new ADAPT procedure able to converge faster to full configuration interaction (FCI).

II. DATA

A. Geometries

TABLE I: Geometries (in Å) of the molecular systems studied present in the paper.

System		x	y	z
N_2	N	0.	0.	0.
	N	0.	0.	1.0977
H_2O	O	0.	0.	0.1173
	H	0.	0.7572	-0.4692
	H	0.	-0.7572	-0.4692
LiH	Li	0.	0.	0.
	H	0.	0.	1.5949
H_n	H	0.	0.	0.0
	H	0.	0.	0.8
	...			
	H	0.	0	$0.8(n-1)$

B. Total ground-state energies with standard basis sets

TABLE II: Ground-state energies (in Ha) for He, Be, FH, LiH, and H_2O at the HF, near-FCI (CIPSI+PT2), ADAPT-VQE (denoted as ADAPT), non-self-consistent basis-set corrected near-FCI (denoted as FCI+PBE+ΔHF), and self-consistent basis-set corrected ADAPT-VQE without the HF basis-set correction (denoted as SC(ADAPT+PBE)). The values of the PBE-based correlation basis-set correction and of the HF basis-set correction are also given. The frozen-core approximation is used for Be, FH, LiH, and H_2O. The CBS limits are estimated by two-point extrapolations from cc-pVQZ and cc-pV5Z calculations. The number of iterations for the ADAPT-VQE calculations are given in bracket.

He	N_{qubits}	HF	ΔHF	PBE	FCI	FCI+PBE+ΔHF	ADAPT	SC(ADAPT+PBE)
STO-3G	2	-2.80778	-0.05384	-0.03182	-2.80778	-2.89344	-	-
pc-seg0	4	-2.83405	-0.02757	-0.01812	-2.84979	-2.89548	-2.84979 [3]	-2.86791
6-31G	4	-2.85516	-0.00646	-0.01950	-2.87016	-2.89612	-2.87016 [3]	-2.88960
cc-pVDZ	10	-2.85516	-0.00646	-0.01168	-2.88759	-2.90573	-2.88759 [6]	-2.89927
cc-pVTZ	28	-2.86115	-0.00047	-0.00420	-2.90023	-2.9049	-	-
cc-pVQZ	60	-2.86151	-0.00011	-0.00167	-2.90241	-2.90419	-	-
cc-pV5Z	110	-2.86162	0	-0.00084	-2.90315	-2.90399	-	-
CBS	-	-	-	-	-2.90392	-	-	-
Be	N_{qubits}	HF	ΔHF	PBE	FCI	FCI+PBE+ΔHF	ADAPT	SC(ADAPT+PBE)
STO-3G	8	-14.35188	-0.22113	-0.00789	-14.40332	-14.63234	-14.40333 [3]	-14.41120
pc-seg0	10	-14.53608	-0.03693	-0.00439	-14.57712	-14.61844	-14.57712 [6]	-14.58160
6-31G	16	-14,56676	-0.00625	-0.00360	-14.61274	-14.62259	-14.61274 [19][a]	-14.61641
cc-pVDZ	26	-14.57234	-0.00067	-0.00187	-14.61684	-14.61938	-14.61684 [24]	-14.61884
cc-pVTZ	58	-14.57287	-0.00014	-0.00082	-14.61842	-14.61938	-	-
cc-pVQZ	108	-14.57297	-0.00004	-0.00038	-14.61895	-14.61937	-	-
cc-pV5Z	180	-14.57301	0	-0.00021	-14.61908	-14.61929	-	-
cc-pV6Z	280	-14.57302	-	-	-	-	-	-
CBS	-	-	-	-	-14.61921	-	-	-
LiH	N_{qubits}	HF	ΔHF	PBE	FCI	FCI+PBE+ΔHF	ADAPT	SC(ADAPT+PBE)
STO-3G	10	-7.86203	-0.12529	-0.01413	-7.88218	-8.0216	-7.88218 [10]	-7.89590
pc-seg0	14	-7.96337	-0.02395	-0.01066	-7.98139	-8.016	-7.98139 [26]	-7.99166
6-31G	20	-7.97927	-0.00805	-0.01063	-7.99800	-8.01668	-7.99800 [47][b]	-8.00806
VQZ-4	10	-7.97603	-0.01129	-0.01344	-7.99281	-8.01754	-7.99281 [10]	-8.00557
V5Z-4	10	-7.97604	-0.01128	-0.01343	-7.99287	-8.01758	-7.99287 [10]	-8.00562
V5Z-7	16	-7.98198	-0.00534	-0.01383	-7.99793	-8.0171	-7.99793 [27]	-8.01658
V5Z-10	28	-7.98326	-0.00406	-0.00867	-8.01302	-8.02575	-8.01302 [91]	-8.02134
cc-pVDZ	36	-7.98373	-0.00359	-0.00418	-8.01438	-8.02215	-	-
cc-pVTZ	86	-7.98665	-0.00067	-0.00145	-8.02234	-8.02446	-	-
cc-pVQZ	190	-7.98718	-0.00014	-0.00063	-8.02386	-8.02463	-	-
cc-pV5Z	290	-7.98732	0	-0.00035	-8.02433	-8.02468	-	-
CBS	-	-	-	-	-8.02482	-	-	-
H_2O	N_{qubits}	HF	ΔHF	PBE	FCI	FCI+PBE+ΔHF	ADAPT	SC(ADAPT+PBE)
STO-3G	12	-74.96302	-1.10403	-0.185798	-75.01250	-76.302328	-75.01250 [63]	-75.19788
pc-seg0	24	-75.77425	-0.2928	-0.135581	-75.90855	-76.336931	-75.90842 [1000][c]	-76.03999
6-31G	24	-75.98397	-0.08308	-0.07738	-76.11995	-76.28041	-76.11989 [1000][d]	-76.23717
V5Z-10	24	-76.01618	-0.05087	-0.15209	-76.12626	-76.32922	-76.12409 [1000]	-76.27418
V5Z-11	30	-76.01756	-0.04948	-0.13591	-76.15902	-76.34441	-76.15165 [541]	-76.28622
cc-pVDZ	46	-76.02677	-0.04028	-0.07406	-76.24165	-76.35599	-	-
cc-pVTZ	114	-76.05713	-0.00992	-0.03063	-76.33250	-76.37305	-	-
cc-pVQZ	228	-76.06479	-0.00226	-0.01471	-76.35985	-76.37682	-	-
cc-pV5Z	400	-76.06705	0	-0.00801	-76.36877	-76.37678	-	-
cc-pV6Z	642	-76.06736	-	-	-	-	-	-
CBS	-	-	-	-	-76.37812	-	-	-
N_2	N_{qubits}	HF	ΔHF	PBE	FCI	FCI+PBE+ΔHF	ADAPT	SC(ADAPT+PBE)
STO-3G	16	-107.49589	-1.49687	-0.21692	-107.65253	-109.36632	-107.65251 [386]	-107.86974
V5Z-6	16	-108.74518	-0.24758	-0.20925	-108.88869	-109.34552	-108.88869 [759]	-109.09850
V5Z-11	32	-108.89413	-0.09896	-0.15816	-109.12739	-109.38451	-109.11566 [916]	-109.27385
cc-pVDZ	52	-108.95412	-0.03864	-0.08984	-109.27698	-109.40546	-	-
cc-pVTZ	116	-108.98347	-0.00929	-0.03695	-109.37527	-109.42151	-	-
cc-pVQZ	216	-108.99108	-0.00168	-0.01788	-109.40558	-109.42514	-	-
cc-pV5Z	360	-108.99276	0	-0.00996	-109.41505	-109.42501	-	-
cc-pV6Z	558	-108.99309					-	-
CBS	-	-	-	-	-109.42498	-	-	-

[a] From the 19th iteration, the ADAPT-VQE iterations kept choosing the same operator, thus the energy stops varying from iteration 19.
[b] From the 47th iteration, the ADAPT-VQE iterations kept choosing the same operator, thus the energy stops varying from iteration 47.
[c] Initial state has 10879 determinants.
[d] Initial state has 14668 determinants.

TABLE III: Ground-state energies (in Ha) for hydrogen chains with atomic distances of 0.8 Å at the HF, near-FCI (CIPSI+PT2), ADAPT-VQE (denoted as ADAPT), non-self-consistent basis-set corrected near-FCI (denoted as FCI+PBE+ΔHF), and self-consistent basis-set corrected ADAPT-VQE without the HF basis-set correction (denoted as SC(ADAPT+PBE)). The values of the PBE-based correlation basis-set correction and of the HF basis-set correction are also given. The CBS limits are estimated by two-point extrapolations from cc-pVQZ and cc-pV5Z calculations. The number of iterations for the ADAPT-VQE calculations are given in bracket.

H_2	N_{qubits}	HF	ΔHF	PBE	FCI	FCI+PBE+ΔHF	ADAPT	SC(ADAPT+PBE)
STO-3G	4	-1.11085	-0.02004	-0.02187	-1.13415	-1.17606	-1.13415 [1]	-1.15590
6-31G	8	-1.12371	-0.00717	-0.01191	-1.15003	-1.16911	-1.15003 [7]	-1.16196
cc-pVDZ	20	-1.12700	-0.00388	-0.00576	-1.16275	-1.17239	-1.16275 [21]	-1.16858
V5Z-8	24	-1.12938	-0.00150	-0.00552	-1.16613	-1.17315	-1.16613 [35]	-1.17170
cc-pVTZ	56	-1.13029	-0.00059	-0.00177	-1.17041	-1.17277	-	-
cc-pVQZ	120	-1.13075	-0.00014	-0.00074	-1.17182	-1.17270	-	-
cc-pV5Z	220	-1.13089	0	-0.00037	-1.17223	-1.17260	-	-
cc-pV6Z	364	-1.13090	-	-	-	-	-	-
CBS	-	-	-	-	-1.17265	-	-	-

H_4	N_{qubits}	HF	ΔHF	PBE	FCI	FCI+PBE+ΔHF	ADAPT	SC(ADAPT+PBE)
STO-3G	8	-2.12139	-0.05672	-0.04299	-2.16756	-2.26727	-2.16756 [19]	-2.21040
cc-pVDZ	40	-2.16785	-0.01026	-0.01188	-2.24884	-2.27098	-	-
cc-pVTZ	112	-2.17696	-0.00115	-0.00371	-2.26848	-2.27333	-	-
cc-pVQZ	240	-2.17782	-0.00029	-0.00157	-2.27134	-2.27320	-	-
cc-pV5Z	438	-2.17811	0	-0.00078	-2.27222	-2.27299	-	-
cc-pV6Z	720	-2.17814	-	-	-	-	-	-
CBS	-	-	-	-	-2.27315	-	-	-

H_6	N_{qubits}	HF	ΔHF	PBE	FCI	FCI+PBE+ΔHF	ADAPT	SC(ADAPT+PBE)
STO-3G	12	-3.13461	-0.09203	-0.04299	-3.20441	-3.33943	-3.20441 [200]	-3.26858
6-31G	24	-3.20987	-0.01677	-0.02653	-3.29582	-3.33912	-	-
cc-pVDZ	60	-3.20989	-0.01675	-0.01801	-3.33763	-3.37239	-	-
cc-pVTZ	168	-3.22483	-0.00181	-0.00566	-3.36939	-3.37686	-	-
cc-pVQZ	360	-3.22619	-0.00045	-0.00241	-3.37379	-3.37665	-	-
cc-pV5Z	650	-3.22664	0	-0.00120	-	-	-	-
cc-pV6Z	1072	-3.22669	-	-	-	-	-	
CBS	-	-	-	-	-	-	-	-

H_8	N_{qubits}	HF	ΔHF	PBE	FCI	FCI+PBE+ΔHF	ADAPT	SC(ADAPT+PBE)
STO-3G	16	-4.14962	-0.1259	-0.08573	-4.24339	-4.45502	-4.24320 [1000]	-4.32764
6-31G	32	-4.25325	-0.02227	-0.05509	-4.37032	-4.44768	-4.35752 [685]	-4.41275
cc-pVDZ	80	-4.25244	-0.02308	-0.02419	-4.42756	-4.47483	-	-
cc-pVTZ	222	-4.27304	-0.00248	-0.00762	-4.47121	-4.48131	-	-
cc-pVQZ	474	-4.27490	-0.00062	-0.00327	-	-	-	-
cc-pV5Z	864	-4.27552	0	-	-	-	-	-
cc-pV6Z	1416	-	-	-	-	-	-	-
CBS	-	-	-	-	-	-	-	-

H_{10}	N_{qubits}	HF	ΔHF	PBE	FCI	FCI+PBE+ΔHF	ADAPT	SC(ADAPT+PBE)
STO-3G	20	-5.16558	-0.15903	-0.10712	-5.28355	-5.5497	-	-
cc-pVDZ	100	-5.29511	-0.0295	-0.03040	-5.51771	-5.57761	-	-
cc-pVTZ	278	-5.32146	-0.00315	-0.00959	-5.57298	-5.58572	-	-
cc-pVQZ	590	-5.32381	-0.0008	-0.00412	-	-	-	-
cc-pV5Z	1074	-5.32461	0	-	-	-	-	-
cc-pV6Z	1758	-	-	-	-	-	-	-
CBS	-	-	-	-	-	-	-	-

C. Total ground-state energies with SABS

The target SABS sizes vary from minimal basis size (STO-3G) to the full size of the original AO basis set.

TABLE IV: [1/2] Ground-state energies (in Ha) of the H_2 molecule calculated by HF, near-FCI (CIPSI+PT2), and self-consistent basis-set corrected near-FCI without and with the HF basis-set correction (denoted as SC(FCI+PBE) and SC(FCI+PBE)+ΔHF, respectively). VXZ-Y corresponds to the cc-pVXZ basis transformed to a basis of Y AO functions following the SABS building procedure. The notation VXZ-[Y_n-Y_{n+a}] means that the values are the same for the basis sets VXY-Y_n, VXY-Y_{n+1}, ..., VXY-Y_{n+a-1}, and VXY-Y_{n+a}. The FCI/CBS limit is -1.13103 Ha.

H_2	Basis set	# active MOs	HF	FCI	SC(FCI+PBE)	SC(FCI+PBE)+ΔHF
	VDZ-[2-3]	2	-1.09635	-1.11372	-1.13412	-1.16880
	VDZ-[4-5]	4	-1.12286	-1.14810	-1.16186	-1.17002
	VDZ-6	10	-1.12700	-1.16275	-1.16857	-1.17260
	VTZ-[2-3]	2	-1.09723	-1.11461	-1.13498	-1.16878
	VTZ-[4-5]	4	-1.12226	-1.14639	-1.16134	-1.17010
	VTZ-[6-7]	6	-1.12513	-1.15191	-1.16293	-1.16883
	VTZ-[8-9]	12	-1.12949	-1.16278	-1.16649	-1.16802
	VTZ-[10-12]	18	-1.13027	-1.16890	-1.17188	-1.17263
	VTZ-13	18	-1.13027	-1.16890	-1.17188	-1.17263
	VTZ-14	28	-1.13029	-1.17040	-1.17215	-1.17289
	VQZ-[2-3]	2	-1.09751	-1.11491	-1.13527	-1.16879
	VQZ-4	4	-1.12096	-1.14421	-1.16002	-1.17008
	VQZ-[5-7]	6	-1.12552	-1.15237	-1.16356	-1.16906
	VQZ-[8-9]	12	-1.13005	-1.16638	-1.17068	-1.17166
	VQZ-10	18	-1.13019	-1.16856	-1.17246	-1.17330
	VQZ-[11-12]	20	-1.13040	-1.16907	-1.17252	-1.17314
	VQZ-[13-15]	26	-1.13067	-1.16987	-1.17214	-1.17250
	VQZ-[16-19]	36	-1.13074	-1.17092	-1.17218	-1.17246
	VQZ-20	46	-1.13074	-1.17158	-1.17258	-1.17286
	VQZ-21	46	-1.13074	-1.17158	-1.17259	-1.17287
	VQZ-22	60	-1.13074	-1.17182	-1.17257	-1.17285
	V5Z-2	2	-1.09759	-1.11499	-1.13535	-1.16879
	V5Z-[3-6]	4	-1.12106	-1.14572	-1.15779	-1.16776
	V5Z-7	10	-1.12579	-1.16014	-1.16560	-1.17083
	V5Z-8	12	-1.12938	-1.16613	-1.17170	-1.17335
	V5Z-9	14	-1.13016	-1.16720	-1.17199	-1.17286
	V5Z-[10-12]	20	-1.13018	-1.16816	-1.17246	-1.17331
	V5Z-[13-15]	26	-1.13078	-1.16986	-1.17232	-1.17256
	V5Z-16	28	-1.13079	-1.17006	-1.17234	-1.17257
	V5Z-17	28	-1.13079	-1.17006	-1.17232	-1.17255
	V5Z-18	38	-1.13081	-1.17101	-1.17290	-1.17312
	V5Z-19	48	-1.13085	-1.17167	-1.17290	-1.17308
	V5Z-20	54	-1.13087	-1.17175	-1.17274	-1.17290
	V5Z-21	54	-1.13087	-1.17175	-1.17274	-1.17289
	V5Z-22	54	-1.13087	-1.17175	-1.17270	-1.17286
	V5Z-[23-26]	68	-1.13087	-1.17194	-1.17281	-1.17296
	V5Z-[27-28]	78	-1.13088	-1.17205	-1.17268	-1.17283
	V5Z-[29-31]	92	-1.13088	-1.17216	-1.17260	-1.17275
	V5Z-32	110	-1.13088	-1.17222	-1.17252	-1.17267

TABLE V: [2/2] Ground-state energies (in Ha) of the H_2 molecule calculated by HF, near-FCI (CIPSI+PT2), and self-consistent basis-set corrected near-FCI without and with the HF basis-set correction (denoted as SC(FCI+PBE) and SC(FCI+PBE)+ΔHF, respectively). VXZ-Y corresponds to the cc-pVXZ basis transformed to a basis of Y AO functions following the SABS building procedure. The notation VXZ-[Y_n-Y_{n+a}] means that the values are the same for the basis sets VXY-Y_n, VXY-Y_{n+1}, ..., VXY-Y_{n+a-1}, and VXY-Y_{n+a}. The FCI/CBS limit is -1.13103 Ha.

H_2	Basis set	# active MOs	HF	FCI	SC(FCI+PBE)	SC(FCI+PBE)+ΔHF
	V6Z-[2-3]	2	-1.09760	-1.11500	-1.13536	-1.16879
	V6Z-4	4	-1.12438	-1.15020	-1.16311	-1.16976
	V6Z-5	10	-1.12791	-1.16391	-1.17072	-1.17383
	V6Z-[6-8]	12	-1.12829	-1.16455	-1.17064	-1.17337
	V6Z-[9-10]	18	-1.12832	-1.16503	-1.17045	-1.17315
	V6Z-11	20	-1.12979	-1.16704	-1.17220	-1.17344
	V6Z-12	26	-1.13068	-1.16963	-1.17239	-1.17273
	V6Z-[13-17]	28	-1.13077	-1.16993	-1.17243	-1.17268
	V6Z-18	38	-1.13081	-1.17129	-1.17301	-1.17323
	V6Z-19	52	-1.13081	-1.17151	-1.17293	-1.17315
	V6Z-20	52	-1.13081	-1.17151	-1.17294	-1.17316
	V6Z-21	54	-1.13082	-1.17156	-1.17292	-1.17313
	V6Z-22	54	-1.13082	-1.17156	-1.17294	-1.17314
	V6Z-23	54	-1.13082	-1.17156	-1.17293	-1.17314
	V6Z-24	60	-1.13087	-1.17173	-1.17276	-1.17291
	V6Z-25	70	-1.13088	-1.17181	-1.17285	-1.17299
	V6Z-26	70	-1.13088	-1.17181	-1.17285	-1.17299
	V6Z-27	80	-1.13089	-1.17203	-1.17270	-1.17283
	V6Z-28	80	-1.13089	-1.17203	-1.17269	-1.17283
	V6Z-29	86	-1.13089	-1.17205	-1.17266	-1.17279
	V6Z-30	86	-1.13089	-1.17205	-1.17267	-1.17280
	V6Z-31	100	-1.13090	-1.17217	-1.17257	-1.17270
	V6Z-[32-33]	100	-1.13090	-1.17217	-1.17268	-1.17281
	V6Z-[34-37]	118	-1.13090	-1.17222	-1.17266	-1.17279
	V6Z-[38-39]	140	-1.13090	-1.17224	-1.17261	-1.17273
	V6Z-[40-45]	150	-1.13090	-1.17226	-1.17256	-1.17269
	V6Z-[46-79]	168	-1.13090	-1.17229	-1.17254	-1.17267

TABLE VI: [1/3] Ground-state energies (in Ha) of the LiH molecule calculated by HF, near-FCI (CIPSI+PT2), and self-consistent basis-set corrected near-FCI without and with the HF basis-set correction (denoted as SC(FCI+PBE) and SC(FCI+PBE)+ΔHF, respectively). VXZ-Y corresponds to the cc-pVXZ basis transformed to a basis of Y AO functions following the SABS building procedure. The notation VXZ-[Y_n-Y_{n+a}] means that the values are the same for the basis sets VXY-Y_n, VXY-Y_{n+1}, ..., VXY-Y_{n+a-1}, and VXY-Y_{n+a}. The FCI/CBS limit is -8.02482 Ha.

LiH	Basis set	# active MOs	HF	FCI	SC(FCI+PBE)	SC(FCI+PBE)+ΔHF
	VDZ-[4-7]	5	-7.97539	-7.99185	-8.00469	-8.01677
	VDZ-8	9	-7.98017	-7.99894	-8.00953	-8.01683
	VDZ-9	12	-7.98147	-8.00858	-8.01321	-8.01922
	VDZ-10	13	-7.98232	-8.01002	-8.01456	-8.01972
	VDZ-11	13	-7.98232	-8.01002	-8.01460	-8.01975
	VDZ-12	18	-7.98372	-8.01437	-8.01851	-8.02226
	VTZ-[4,5]	5	-7.97596	-7.99267	-8.00544	-8.01696
	VTZ-[6-8]	8	-7.98028	-7.99634	-8.00932	-8.01652
	VTZ-9	11	-7.98340	-7.99981	-8.01260	-8.01668
	VTZ-10	12	-7.98447	-8.00140	-8.01392	-8.01693
	VTZ-11	17	-7.98509	-8.00438	-8.01549	-8.01788
	VTZ-12	20	-7.98592	-8.01781	-8.02355	-8.02510
	VTZ-[13-15]	21	-7.98606	-8.01837	-8.02278	-8.02420
	VTZ-16	26	-7.98627	-8.01898	-8.02319	-8.02440
	VTZ-[17,18]	33	-7.98643	-8.01973	-8.02368	-8.02473
	VTZ-19	38	-7.98647	-8.02061	-8.02286	-8.02386
	VTZ-[20-23]	41	-7.98652	-8.02151	-8.02302	-8.02397
	VTZ-[24-41]	42	-7.98661	-8.02212	-8.02359	-8.02446
	VTZ-42	43	-7.98664	-8.02234	-8.02378	-8.02461
	VQZ-[4,5]	5	-7.97603	-7.99281	-8.00557	-8.01702
	VQZ-[6-9]	8	-7.97985	-7.99605	-8.00895	-8.01657
	VQZ-[10,11]	12	-7.98304	-8.00059	-8.01089	-8.01532
	VQZ-12	15	-7.98404	-8.01500	-8.02051	-8.02394
	VQZ-[13,14]	16	-7.98469	-8.01585	-8.02132	-8.02411
	VQZ-15	21	-7.98561	-8.01772	-8.02277	-8.02463
	VQZ-16	22	-7.98575	-8.01817	-8.02285	-8.02458
	VQZ-17	25	-7.98651	-8.01900	-8.02364	-8.02461
	VQZ-18	30	-7.98659	-8.02043	-8.02331	-8.02419
	VQZ-[19-21]	35	-7.98661	-8.02065	-8.02345	-8.02432
	VQZ-22	36	-7.98696	-8.02107	-8.02385	-8.02437
	VQZ-23	39	-7.98705	-8.02276	-8.02443	-8.02485
	VQZ-[24,25]	44	-7.98712	-8.02288	-8.02448	-8.02483
	VQZ-[26-28]	51	-7.98714	-8.02316	-8.02473	-8.02507
	VQZ-[29,30]	58	-7.98714	-8.02326	-8.02478	-8.02511
	VQZ-31	61	-7.98715	-8.02341	-8.02455	-8.02488
	VQZ-32	62	-7.98715	-8.02350	-8.02462	-8.02494
	VQZ-33	63	-7.98716	-8.02357	-8.02468	-8.02500
	VQZ-34	72	-7.98717	-8.02366	-8.02472	-8.02503
	VQZ-[35-38]	79	-7.98717	-8.02381	-8.02458	-8.02489
	VQZ-39	84	-7.98717	-8.02391	-8.02453	-8.02484

TABLE VII: [2/3] Ground-state energies (in Ha) of the LiH molecule calculated by HF, near-FCI (CIPSI+PT2), and self-consistent basis-set corrected near-FCI without and with the HF basis-set correction (denoted as SC(FCI+PBE) and SC(FCI+PBE)+ΔHF, respectively). VXZ-Y corresponds to the cc-pVXZ basis transformed to a basis of Y AO functions following the SABS building procedure. The notation VXZ-[Y_n-Y_{n+a}] means that the values are the same for the basis sets VXY-Y_n, VXY-Y_{n+1}, ..., VXY-Y_{n+a-1}, and VXY-Y_{n+a}. The FCI/CBS limit is -8.02482 Ha.

LiH	Basis set	# active MOs	HF	FCI	SC(FCI+PBE)	SC(FCI+PBE)+ΔHF
	V5Z-[4-6]	5	-7.97604	-7.99287	-8.00561	-8.01705
	V5Z-[7,8]	8	-7.98198	-7.99793	-8.01108	-8.01657
	V5Z-9	11	-7.98238	-7.99900	-8.01179	-8.01689
	V5Z-10	14	-7.98326	-8.01302	-8.02133	-8.02554
	V5Z-[11,12]	15	-7.98391	-8.01389	-8.02195	-8.02552
	V5Z-13	16	-7.98420	-8.01421	-8.02227	-8.02555
	V5Z-14	19	-7.98543	-8.01533	-8.02336	-8.02541
	V5Z-15	20	-7.98630	-8.01710	-8.02423	-8.02541
	V5Z-16	21	-7.98651	-8.01757	-8.02375	-8.02471
	V5Z-17	22	-7.98657	-8.01778	-8.02382	-8.02472
	V5Z-[18,19]	27	-7.98673	-8.01954	-8.02377	-8.02452
	V5Z-20	32	-7.98673	-8.01970	-8.02385	-8.02460
	V5Z-[21,22]	35	-7.98683	-8.02212	-8.02454	-8.02519
	V5Z-23	38	-7.98714	-8.02247	-8.02487	-8.02520
	V5Z-[24,25]	39	-7.98724	-8.02298	-8.02493	-8.02517
	V5Z-26	44	-7.98724	-8.02317	-8.02506	-8.02529
	V5Z-[27-29]	49	-7.98728	-8.02326	-8.02510	-8.02530
	V5Z-[30-32]	56	-7.98728	-8.02338	-8.02517	-8.02536
	V5Z-33	60	-7.98729	-8.02362	-8.02499	-8.02517
	V5Z-34	67	-7.98730	-8.02384	-8.02484	-8.02502
	V5Z-35	68	-7.98732	-8.02387	-8.02487	-8.02503
	V5Z-36	68	-7.98732	-8.02387	-8.02485	-8.02501
	V5Z-[37-39]	75	-7.98732	-8.02389	-8.02487	-8.02503
	V5Z-40	78	-7.98732	-8.02390	-8.02472	-8.02488
	V5Z-41	83	-7.98732	-8.02403	-8.02465	-8.02481
	V5Z-42	90	-7.98732	-8.02412	-8.02453	-8.02469
	V5Z-43	90	-7.98732	-8.02412	-8.02455	-8.02471
	V5Z-44	90	-7.98732	-8.02412	-8.02448	-8.02464
	V5Z-45	90	-7.98732	-8.02412	-8.02452	-8.02467
	V5Z-46	90	-7.98732	-8.02408	-8.02469	-8.02484
	V5Z-47	99	-7.98732	-8.02406	-8.02466	-8.02481
	V5Z-48	99	-7.98732	-8.02413	-8.02472	-8.02488
	V5Z-49	99	-7.98732	-8.02412	-8.02472	-8.02487
	V5Z-50	99	-7.98732	-8.02413	-8.02468	-8.02483
	V5Z-51	99	-7.98732	-8.02409	-8.02470	-8.02486
	V5Z-52	106	-7.98732	-8.02415	-8.02466	-8.02482
	V5Z-53	115	-7.98732	-8.02420	-8.02443	-8.02459
	V5Z-54	115	-7.98732	-8.02420	-8.02452	-8.02468
	V5Z-55	115	-7.98732	-8.02420	-8.02452	-8.02468
	V5Z-56	120	-7.98732	-8.02427	-8.02451	-8.02466
	V5Z-57	129	-7.98732	-8.02430	-8.02445	-8.02461
	V5Z-58	129	-7.98732	-8.02430	-8.02449	-8.02464
	V5Z-59	129	-7.98732	-8.02430	-8.02450	-8.02465
	V5Z-60	129	-7.98732	-8.02430	-8.02448	-8.02464

TABLE VIII: [3/3] Ground-state energies (in Ha) of the LiH molecule calculated by HF, near-FCI (CIPSI+PT2), and self-consistent basis-set corrected near-FCI without and with the HF basis-set correction (denoted as SC(FCI+PBE) and SC(FCI+PBE)+ΔHF, respectively). VXZ-Y corresponds to the cc-pVXZ basis transformed to a basis of Y AO functions following the SABS building procedure. The notation VXZ-[Y_n-Y_{n+a}] means that the values are the same for the basis sets VXY-Y_n, VXY-Y_{n+1}, ..., VXY-Y_{n+a-1}, and VXY-Y_{n+a}. The FCI/CBS limit is -8.02482 Ha.

LiH	Basis set	# active MOs	HF	FCI	SC(FCI+PBE)	SC(FCI+PBE)+ΔHF
	V5Z-61	129	-7.98732	-8.02430	-8.02450	-8.02466
	V5Z-62	129	-7.98732	-8.02430	-8.02454	-8.02469
	V5Z-63	140	-7.98732	-8.02432	-8.02442	-8.02458
	V5Z-64	140	-7.98732	-8.02432	-8.02446	-8.02462
	V5Z-65	140	-7.98732	-8.02432	-8.02430	-8.02445
	V5Z-66	140	-7.98732	-8.02432	-8.02449	-8.02464
	V5Z-67	140	-7.98732	-8.02432	-8.02435	-8.02451
	V5Z-68	140	-7.98732	-8.02432	-8.02447	-8.02462
	V5Z-69	140	-7.98732	-8.02432	-8.02433	-8.02449
	V5Z-70	140	-7.98732	-8.02432	-8.02425	-8.02440
	V5Z-71	140	-7.98732	-8.02432	-8.02428	-8.02443
	V5Z-72	140	-7.98732	-8.02432	-8.02437	-8.02453
	V5Z-73	140	-7.98732	-8.02432	-8.02451	-8.02467
	V5Z-74	140	-7.98732	-8.02432	-8.02432	-8.02448
	V5Z-75	140	-7.98732	-8.02432	-8.02426	-8.02441
	V5Z-76	140	-7.98732	-8.02432	-8.02440	-8.02456
	V5Z-77	140	-7.98732	-8.02432	-8.02430	-8.02445
	V5Z-78	140	-7.98732	-8.02432	-8.02428	-8.02444
	V5Z-79	140	-7.98732	-8.02432	-8.02433	-8.02449
	V5Z-80	140	-7.98732	-8.02432	-8.02431	-8.02447
	V5Z-81	145	-7.98732	-8.02431	-8.02399	-8.02415

TABLE IX: [1/2] Ground-state energies (in Ha) of the H_2O molecule calculated by HF, near-FCI (CIPSI+PT2), and self-consistent basis-set corrected near-FCI without and with the HF basis-set correction (denoted as SC(FCI+PBE) and SC(FCI+PBE)+ΔHF, respectively). VXZ-Y corresponds to the cc-pVXZ basis transformed to a basis of Y AO functions following the SABS building procedure. The notation VXZ-[Y_n-Y_{n+a}] means that the values are the same for the basis sets VXY-Y_n, VXY-Y_{n+1}, ..., VXY-Y_{n+a-1}, and VXY-Y_{n+a}. The FCI/CBS limit is -76.37812 Ha.

H_2O	Basis set	# active MOs	HF	FCI	SC(FCI+PBE)	SC(FCI+PBE)+ΔHF
	VDZ-5	6	-75.89241	-75.93669	-76.11929	-76.29628
	VDZ-6	7	-75.90086	-75.95934	-76.13269	-76.30124
	VDZ-7	9	-75.96623	-76.02968	-76.19811	-76.30128
	VDZ-8	9	-75.96623	-76.02968	-76.19811	-76.30128
	VDZ-9	12	-75.98044	-76.11620	-76.25492	-76.34389
	VDZ-10	12	-75.98044	-76.11620	-76.25499	-76.34396
	VDZ-11	18	-76.01858	-76.17283	-76.29347	-76.34430
	VDZ-12	18	-76.01858	-76.17283	-76.29347	-76.34430
	VDZ-13	18	-76.01858	-76.17283	-76.29347	-76.34430
	VDZ-14	18	-76.01858	-76.17283	-76.29347	-76.34430
	VDZ-15	23	-76.02677	-76.24164	-76.31537	-76.35801
	VTZ-5	6	-75.90857	-75.95344	-76.13540	-76.29623
	VTZ-6	6	-75.90857	-75.95344	-76.13540	-76.29623
	VTZ-7	8	-75.97635	-76.02689	-76.20462	-76.29768
	VTZ-8	8	-75.97635	-76.02689	-76.20461	-76.29767
	VTZ-9	11	-75.99693	-76.11476	-76.26502	-76.33749
	VTZ-10	12	-76.00181	-76.13304	-76.27671	-76.34430
	VTZ-11	12	-76.00181	-76.13304	-76.27661	-76.34420
	VTZ-12	18	-76.03306	-76.17994	-76.31259	-76.34894
	VTZ-13	20	-76.03462	-76.18539	-76.31508	-76.34987
	VTZ-14	20	-76.03462	-76.18539	-76.31508	-76.34987
	VTZ-15	20	-76.03462	-76.18539	-76.31508	-76.34987
	VTZ-16	26	-76.04399	-76.20157	-76.31989	-76.34530
	VTZ-17	31	-76.05250	-76.25599	-76.34643	-76.36333
	VTZ-18	31	-76.05250	-76.25599	-76.34643	-76.36333
	VTZ-19	36	-76.05538	-76.28894	-76.34552	-76.35954
	VTZ-20	39	-76.05554	-76.30272	-76.35240	-76.36626
	VTZ-21	39	-76.05554	-76.30298	-76.35240	-76.36626
	VTZ-22	49	-76.05664	-76.31067	-76.35578	-76.36854
	VTZ-23	56	-76.05705	-76.32636	-76.36003	-76.37239
	VQZ-5	6	-75.91275	-75.95778	-76.13974	-76.29640
	VQZ-6	6	-75.91275	-75.95778	-76.13974	-76.29640
	VQZ-7	6	-75.91275	-75.95778	-76.13974	-76.29640
	VQZ-8	8	-75.97626	-76.02635	-76.20482	-76.29796
	VQZ-9	11	-75.99855	-76.11074	-76.26425	-76.33510
	VQZ-10	12	-76.00570	-76.12947	-76.27719	-76.34089
	VQZ-11	14	-76.02138	-76.14741	-76.29062	-76.33865
	VQZ-12	14	-76.02138	-76.14741	-76.29062	-76.33865
	VQZ-13	20	-76.04618	-76.19039	-76.31636	-76.33958
	VQZ-14	20	-76.04618	-76.19039	-76.31636	-76.33958
	VQZ-15	23	-76.04840	-76.21652	-76.32652	-76.34753
	VQZ-16	28	-76.05728	-76.28404	-76.34910	-76.36123
	VQZ-17	34	-76.05950	-76.29193	-76.35589	-76.36579
	VQZ-18	39	-76.06046	-76.30063	-76.36204	-76.37098
	VQZ-19	39	-76.06046	-76.30063	-76.36205	-76.37099
	VQZ-20	39	-76.06046	-76.30065	-76.36204	-76.37098
	VQZ-21	45	-76.06207	-76.30361	-76.36256	-76.36990
	VQZ-22	45	-76.06207	-76.30361	-76.36256	-76.36990
	VQZ-23	50	-76.06247	-76.31192	-76.35916	-76.36609

TABLE X: [2/2] Ground-state energies (in Ha) of the H_2O molecule calculated by HF, near-FCI (CIPSI+PT2), and self-consistent basis-set corrected near-FCI without and with the HF basis-set correction (denoted as SC(FCI+PBE) and SC(FCI+PBE)+ΔHF, respectively). VXZ-Y corresponds to the cc-pVXZ basis transformed to a basis of Y AO functions following the SABS building procedure. The notation VXZ-[Y_n-Y_{n+a}] means that the values are the same for the basis sets VXY-Y_n, VXY-Y_{n+1}, ..., VXY-Y_{n+a-1}, and VXY-Y_{n+a}. The FCI/CBS limit is -76.37812 Ha.

H_2O	Basis set	# active MOs	HF	FCI	SC(FCI+PBE)	SC(FCI+PBE)+ΔHF
	V5Z-5	6	-75.91383	-75.95891	-76.14081	-76.29638
	V5Z-6	6	-75.91383	-75.95891	-76.14081	-76.29638
	V5Z-7	6	-75.91383	-75.95891	-76.14081	-76.29638
	V5Z-8	8	-75.99384	-76.04603	-76.22026	-76.29583
	V5Z-9	9	-76.00422	-76.07508	-76.23959	-76.30477
	V5Z-10	12	-76.01618	-76.12626	-76.27636	-76.32958
	V5Z-11	15	-76.01756	-76.15902	-76.29319	-76.34504
	V5Z-12	15	-76.01756	-76.15902	-76.29321	-76.34506
	V5Z-13	17	-76.02137	-76.16455	-76.29813	-76.34616
	V5Z-14	23	-76.05007	-76.21406	-76.33178	-76.35112
	V5Z-15	28	-76.05818	-76.27612	-76.35656	-76.36779
	V5Z-16	28	-76.05818	-76.27612	-76.35656	-76.36779
	V5Z-17	30	-76.05939	-76.27878	-76.35751	-76.36752
	V5Z-18	30	-76.05939	-76.27878	-76.35751	-76.36752
	V5Z-19	30	-76.05939	-76.27878	-76.35751	-76.36752
	V5Z-20	30	-76.05939	-76.27878	-76.35751	-76.36752
	V6Z-5	6	-75.91390	-75.95899	-76.14088	-76.29639
	V6Z-6	6	-75.91390	-75.95899	-76.14088	-76.29639
	V6Z-7	6	-75.91390	-75.95899	-76.14088	-76.29639
	V6Z-8	8	-75.99599	-76.04790	-76.22379	-76.29720
	V6Z-9	9	-76.00502	-76.07392	-76.24098	-76.30537
	V6Z-10	12	-76.01665	-76.14766	-76.29046	-76.34322
	V6Z-11	18	-76.04304	-76.19235	-76.32124	-76.34761
	V6Z-12	18	-76.04304	-76.19233	-76.32127	-76.34763
	V6Z-13	21	-76.04482	-76.19875	-76.32480	-76.34939
	V6Z-14	22	-76.04584	-76.20378	-76.32573	-76.34930
	V6Z-15	24	-76.04619	-76.20739	-76.32728	-76.35050
	V6Z-16	24	-76.04619	-76.20740	-76.32728	-76.35050
	V6Z-17	29	-76.05504	-76.26201	-76.35272	-76.36709
	V6Z-18	32	-76.05517	-76.27309	-76.35519	-76.36943
	V6Z-19	38	-76.05960	-76.28117	-76.35808	-76.36789
	V6Z-20	38	-76.05960	-76.28166	-76.35808	-76.36789

TABLE XI: Ground-state energies (in Ha) of the N_2 molecule calculated by HF, near-FCI (CIPSI+PT2), and self-consistent basis-set corrected near-FCI without and with the HF basis-set correction (denoted as SC(FCI+PBE) and SC(FCI+PBE)+ΔHF, respectively). VXZ-Y corresponds to the cc-pVXZ basis transformed to a basis of Y AO functions following the SABS building procedure. The notation VXZ-[Y_n-Y_{n+a}] means that the values are the same for the basis sets VXY-Y_n, VXY-Y_{n+1}, ..., VXY-Y_{n+a-1}, and VXY-Y_{n+a}. The FCI/CBS limit is -109.42498 Ha.

N_2	Basis set	# active MOs	HF	FCI	SC(FCI+PBE)	SC(FCI+PBE)+ΔHF
	VQZ-10	14	-108.82485	-109.01533	-109.20135	-109.37101
	VQZ-11	16	-108.88278	-109.10163	-109.27284	-109.38458
	VQZ-[12-14]	22	-108.90058	-109.14692	-109.29652	-109.39046
	VQZ-[15-16]	24	-108.90226	-109.15252	-109.29290	-109.38516
	VQZ-[17-20]	34	-108.98089	-109.31664	-109.39284	-109.40647
	VQZ-[21-25]	44	-108.98341	-109.32951	-109.38867	-109.39977
	VQZ-26	58	-108.98412	-109.35139	-109.39949	-109.40989
	VQZ-27	68	-108.98955	-109.36564	-109.41443	-109.41940
	V5Z-10	14	-108.80656	-109.01099	-109.18262	-109.37057
	V5Z-[11-13]	16	-108.89413	-109.12739	-109.28517	-109.38556
	V5Z-14	26	-108.97053	-109.28372	-109.38187	-109.40585
	V5Z-[15-17]	32	-108.97450	-109.29460	-109.39378	-109.41380
	V5Z-18	34	-108.97679	-109.30157	-109.39547	-109.41320
	V5Z-19	40	-108.97763	-109.31094	-109.39763	-109.41451

D. Dissociation curves

TABLE XII: Ground-state energies (in Ha) used for the dissociation curves of H_2 at the HF, near-FCI (CIPSI+PT2), self-consistent basis-set corrected ADAPT-VQE without the HF basis-set correction (denoted as SC(A+PBE)), and with the HF basis-set correction (denoted as SC(A+PBE)+ΔHF). Distances are in Å. VXZ stands for the standard cc-pVXZ basis set.

Basis set	Distance	HF	FCI	SC(A+PBE)	SC(A+PBE)+ΔHF
VDZ	0.5	-1.04880	-1.07937	-1.08802	-1.10414
	0.6	-1.10689	-1.13917	-1.14667	-1.15616
	0.7	-1.12692	-1.16090	-1.16748	-1.17333
	0.8	-1.12700	-1.16275	-1.16858	-1.17246
	0.9	-1.11639	-1.15408	-1.15930	-1.16218
	1.0	-1.10015	-1.14007	-1.14481	-1.14724
	1.5	-1.00219	-1.06153	-1.06513	-1.06787
	2.0	-0.92191	-1.01759	-1.02089	-1.02522
	2.5	-0.86533	-1.00313	-1.00631	-1.01238
	3.0	-0.82645	-0.99955	-1.00268	-1.01043
V5Z-8	0.5	-1.05683	-1.08907	-1.09726	-1.10535
	0.6	-1.11193	-1.14569	-1.15284	-1.15729
	0.7	-1.13028	-1.16548	-1.17178	-1.17428
	0.8	-1.12938	-1.16614	-1.17170	-1.17321
	0.9	-1.11825	-1.15679	-1.16174	-1.16276
	1.0	-1.10177	-1.14243	-1.14689	-1.14770
	1.5	-1.00378	-1.06363	-1.06675	-1.06791
	2.0	-0.92434	-1.01944	-1.02219	-1.02409
	2.5	-0.86969	-1.00467	-1.00739	-1.00911
	3.0	-0.83308	-1.00100	-1.00373	-1.00484
VTZ	0.5	-	-1.10087	-	-
	0.6	-	-1.15352	-	-
	0.7	-	-1.17101	-	-
	0.8	-	-1.17041	-	-
	0.9	-	-1.16041	-	-
	1.0	-	-1.14576	-	-
	1.5	-	-1.06617	-	-
	2.0	-	-1.02046	-	-
	2.5	-	-1.00467	-	-
	3.0	-	-1.00072	-	-
V5Z	0.5	-1.06492	-1.10397	-	-
	0.6	-1.11638	-1.15581	-	-
	0.7	-1.13278	-1.17297	-	-
	0.8	-1.13089	-1.17223	-	-
	0.9	-1.11927	-1.16216	-	-
	1.0	-1.10258	-1.14748	-	-
	1.5	-1.00493	-1.06795	-	-
	2.0	-0.92624	-1.02186	-	-
	2.5	-0.87140	-1.00550	-	-
	3.0	-0.83420	-1.00124	-	-

TABLE XIII: Ground-state energies (in Ha) used for the dissociation curves of LiH at the HF, near-FCI (CIPSI+PT2), self-consistent basis-set corrected ADAPT-VQE without the HF basis-set correction (denoted as SC(A+PBE)), and with the HF basis-set correction (denoted as SC(A+PBE)+ΔHF). Distances are in Å. VXZ stands for the standard cc-pVXZ basis set.

Basis set	Distance	HF	FCI	SC(A+PBE)	SC(A+PBE)+ΔHF
6-31G	0.5	-7.14721	-7.16953	-7.17775	-7.23149
	0.75	-7.66281	-7.68441	-7.69458	-7.72292
	1.0	-7.87136	-7.89039	-7.90091	-7.91794
	1.25	-7.95144	-7.96925	-7.97975	-7.99182
	1.5	-7.97686	-7.99510	-8.00533	-8.01428
	1.75	-7.97824	-7.99809	-8.00780	-8.01473
	2.0	-7.96887	-7.99121	-8.00025	-8.00617
	2.25	-7.95492	-7.98053	-7.98884	-7.99461
	2.5	-7.93936	-7.96907	-7.97663	-7.98291
	2.75	-7.92365	-7.95844	-7.96522	-7.97253
	3.0	-7.90850	-7.94951	-7.95550	-7.96420
V5Z-7	0.5	-7.10996	-7.13045	-7.14080	-7.23179
	0.75	-7.65310	-7.66747	-7.68139	-7.71943
	1.0	-7.87189	-7.88480	-7.89919	-7.91570
	1.25	-7.95443	-7.96789	-7.98184	-7.99092
	1.5	-7.97979	-7.99488	-8.00827	-8.01429
	1.75	-7.98055	-7.99819	-8.01091	-8.01554
	2.0	-7.97042	-7.99152	-8.00347	-8.00784
	2.25	-7.95565	-7.98110	-7.99226	-7.99729
	2.5	-7.93924	-7.97003	-7.98041	-7.98681
	2.75	-7.92265	-7.95988	-7.96953	-7.97783
	3.0	-7.90660	-7.95145	-7.96043	-7.97104
VDZ	0.5	-	-7.19664	-	-
	0.75	-	-7.71273	-	-
	1.0	-	-7.91535	-	-
	1.25	-	-7.99037	-	-
	1.5	-	-8.01264	-	-
	1.75	-	-8.01278	-	-
	2.0	-	-8.00361	-	-
	2.25	-	-7.99108	-	-
	2.5	-	-7.97809	-	-
	2.75	-	-7.96613	-	-
	3.0	-	-7.95594	-	-
V5Z	0.5	-7.20095	-7.24405	-	-
	0.75	-7.69115	-7.73130	-	-
	1.0	-7.88839	-7.92707	-	-
	1.25	-7.96350	-8.00106	-	-
	1.5	-7.98581	-8.02285	-	-
	1.75	-7.98517	-8.02236	-	-
	2.0	-7.97479	-8.01280	-	-
	2.25	-7.96068	-8.00003	-	-
	2.5	-7.94564	-7.98672	-	-
	2.75	-7.93096	-7.97443	-	-
	3.0	-7.91720	-7.96344	-	-

TABLE XIV: Ground-state energies (in Ha) used for the dissociation curves of N_2 at the HF, near-FCI (CIPSI+PT2), self-consistent basis-set corrected ADAPT-VQE without the HF basis-set correction (denoted as SC(A+PBE)), and with the HF basis-set correction (denoted as SC(A+PBE)+ΔHF). Distances are in Å. VXZ stands for the standard cc-pVXZ basis set.

Basis set	Distance	HF	FCI	SC(A+PBE)	SC(A+PBE)+ΔHF
STO-3G	0.8	-106.68080	-106.76594	-106.99161	-108.82882
	0.9	-107.18719	-107.29271	-107.51568	-109.18175
	1.0977	-107.49589	-107.65251	-107.86975	-109.36662
	1.2	-107.48778	-107.67707	-107.88963	-109.35016
	1.5	-107.27245	-107.58147	-107.78293	-109.22200
	2.0	-106.87150	-107.45512	-107.65901	-109.15567
	2.5	-106.61696	-107.44041	-107.63628	-109.18056
V5Z-6	0.8	-107.89375	-107.97171	-108.19082	-108.81508
	0.9	-108.39476	-108.49175	-108.70783	-109.16634
	1.0977	-108.74519	-108.88869	-109.09851	-109.34608
	1.2	-108.76756	-108.93985	-109.14120	-109.32195
	1.5	-108.63207	-108.90483	-109.09718	-109.17662
	2	-108.32055	-108.81060	-109.00808	-109.05568
	2.5	-108.10572	-108.80176	-109.00012	-109.05564
VDZ	0.8	-	-108.66035	-	-
	0.9	-	-109.05746	-	-
	1.1	-	-109.27657	-	-
	1.2	-	-109.26410	-	-
	1.5	-	-109.12459	-	-
	2.0	-	-108.98115	-	-
	2.5	-	-108.95868	-	-
VTZ	0.8	-	-108.82771	-	-
	0.9	-	-109.18511	-	-
	1.1	-	-109.36884	-	-
	1.2	-	-109.34807	-	-
	1.5	-	-109.20218	-	-
	2.0	-	-109.04838	-	-
	2.5	-	-109.01953	-	-
V5Z	0.8	-108.51801	-	-	-
	0.9	-108.85326	-	-	-
	1.0977	-108.99276	-	-	-
	1.2	-108.94831	-	-	-
	1.5	-108.71151	-	-	-
	2.0	-108.36816	-	-	-
	2.5	-108.16125	-	-	-

E. Dipole moments

TABLE XV: Dipole moments (in atomic units) of LiH and H_2O computed as expectation values of HF and near-FCI (CIPSI) wave functions, and the near-FCI dipole moments with the HF basis-set correction $\Delta\text{HF} = d_{\text{HF}}^{\text{aug-cc-pV5Z}} - d_{\text{HF}}^{\mathcal{B}}$, where $d_{\text{HF}}^{\text{aug-cc-pV5Z}}$ is the aug-cc-pV5Z HF dipole moment and $d_{\text{HF}}^{\mathcal{B}}$ is the HF dipole moment in the basis set $\mathcal{B}$ considered. aug-cc-pVXZ values are provided for informational purposes.

LiH	N_{qubits}	HF	FCI	FCI + ΔHF
STO-3G	10	-1.91107	-1.81835	-2.26857
pcseg-0	14	-2.47768	-2.33313	-2.21674
6-31G	20	-2.33223	-2.16646	-2.19552
V5Z-4	10	-2.57388	-2.37818	-2.16559
V5Z-7	16	-2.38299	-2.23095	-2.20925
V5Z-10	28	-2.32149	-2.24997	-2.28977
cc-pVDZ	36	-2.33994	-2.25566	-2.27701
cc-pVTZ	86	-2.35488	-2.29998	-2.30639
cc-pVQZ	190	-2.35762	-2.30361	-2.30728
cc-pV5Z	290	-2.36017	-2.30647	-2.30759
aug-cc-pVDZ	62	-2.37055	-2.32496	-2.3157
aug-cc-pVTZ	136	-2.36235	-2.31028	-2.30922
aug-cc-pVQZ	250	-2.36152	-2.30792	-2.30769
aug-cc-pV5Z	412	-2.36129	-2.30787	-2.30787
H_2O	N_{qubits}	HF	FCI	FCI + ΔHF
STO-3G	12	-0.67878	-0.63584	-0.73662
pcseg-0	24	-1.00341	-0.95822	-0.73437
6-31G	24	-1.03512	-0.9902	-0.73464
V5Z-10	24	-1.02208	-0.99305	-0.75053
V5Z-11	30	-1.02389	-0.99185	-0.74752
cc-pVDZ	46	-0.80943	-0.76073	-0.73086
cc-pVTZ	114	-0.79709	-0.74858	-0.73105
cc-pVQZ	228	-0.79006	-0.74409	-0.73359
cc-pV5Z	400	-0.78783	-0.74241	-0.73414
aug-cc-pVDZ	80	-0.78671	-0.72703[a]	-0.71988[a]
aug-cc-pVTZ	181	-0.78038	-0.72364[a]	-0.72282[a]
aug-cc-pVQZ	342	-0.77955	-0.72695[a]	-0.72696[a]
aug-cc-pV5Z	572	-0.77956	-0.72815[a]	-0.72815[a]

[a] CCSD(T) values from Ref. [13].

III. SABS

In this section, we give the basis functions in our SABS used in Table II of the paper.

A. H_2, SABS/V5Z-8

```
HYDROGEN
S 8
    1  4.02E+02   2.79E-04
    2  6.02E+01   2.17E-03
    3  1.37E+01   1.12E-02
    4  3.91E+00   4.49E-02
    5  1.28E+00   1.42E-01
    6  4.66E-01   3.31E-01
    7  1.81E-01   4.36E-01
    8  7.28E-02   1.76E-01
S 1
    1  1.81E-01   1.00E+00
S 1
    1  7.28E-02   1.00E+00
P 1
    1  6.49E-01   1.00E+00
```

B. LiH, SABS/V5Z-4

```
LITHIUM
S 14
 1 2.949300E+04  1.800000E-05
 2 4.417101E+03  1.410000E-04
 3 1.005223E+03  7.390000E-04
 4 2.847009E+02  3.107000E-03
 5 9.286543E+01  1.113500E-02
 6 3.351179E+01  3.467000E-02
 7 1.304180E+01  9.217100E-02
 8 5.357536E+00  1.995760E-01
 9 2.279338E+00  3.288360E-01
10 9.939900E-01  3.459750E-01
11 4.334710E-01  1.427610E-01
12 9.556600E-02  5.319000E-03
13 4.465700E-02  -2.101000E-03
14 2.063300E-02  8.150000E-04
S 14
 1 2.949300E+04  -3.000000E-06
 2 4.417101E+03  -2.200000E-05
 3 1.005223E+03  -1.150000E-04
 4 2.847009E+02  -4.870000E-04
 5 9.286543E+01  -1.746000E-03
 6 3.351179E+01  -5.520000E-03
 7 1.304180E+01  -1.492800E-02
 8 5.357536E+00  -3.420600E-02
 9 2.279338E+00  -6.215500E-02
10 9.939900E-01  -9.590200E-02
11 4.334710E-01  -1.039720E-01
12 9.556600E-02  3.071510E-01
13 4.465700E-02  5.790280E-01
14 2.063300E-02  2.232310E-01
P 8
 1 1.966350E+01  5.400000E-04
 2 4.623110E+00  3.865000E-03
 3 1.413780E+00  1.517100E-02
 4 4.737210E-01  4.920400E-02
 5 1.761510E-01  1.546610E-01
 6 7.267500E-02  3.600950E-01
 7 3.214100E-02  4.418520E-01
 8 1.455600E-02  1.560000E-01
HYDROGEN
S 8
 1 4.020000E+02  2.790000E-04
 2 6.024000E+01  2.165000E-03
 3 1.373000E+01  1.120100E-02
 4 3.905000E+00  4.487800E-02
 5 1.283000E+00  1.422990E-01
 6 4.655000E-01  3.309790E-01
 7 1.811000E-01  4.362690E-01
 8 7.279000E-02  1.764400E-01
```

C. LiH, SABS/V5Z-7

```
LITHIUM
S 14
          1 2.949300E+04  1.800000E-05
          2 4.417101E+03  1.410000E-04
          3 1.005223E+03  7.390000E-04
          4 2.847009E+02  3.107000E-03
          5 9.286543E+01  1.113500E-02
          6 3.351179E+01  3.467000E-02
          7 1.304180E+01  9.217100E-02
          8 5.357536E+00  1.995760E-01
          9 2.279338E+00  3.288360E-01
         10 9.939900E-01  3.459750E-01
         11 4.334710E-01  1.427610E-01
         12 9.556600E-02  5.319000E-03
         13 4.465700E-02  -2.101000E-03
         14 2.063300E-02  8.150000E-04
S 14
          1 2.949300E+04  -3.000000E-06
          2 4.417101E+03  -2.200000E-05
          3 1.005223E+03  -1.150000E-04
          4 2.847009E+02  -4.870000E-04
          5 9.286543E+01  -1.746000E-03
          6 3.351179E+01  -5.520000E-03
          7 1.304180E+01  -1.492800E-02
          8 5.357536E+00  -3.420600E-02
          9 2.279338E+00  -6.215500E-02
         10 9.939900E-01  -9.590200E-02
         11 4.334710E-01  -1.039720E-01
         12 9.556600E-02  3.071510E-01
         13 4.465700E-02  5.790280E-01
         14 2.063300E-02  2.232310E-01
P 8
          1 1.966350E+01  5.400000E-04
          2 4.623110E+00  3.865000E-03
          3 1.413780E+00  1.517100E-02
          4 4.737210E-01  4.920400E-02
          5 1.761510E-01  1.546610E-01
          6 7.267500E-02  3.600950E-01
          7 3.214100E-02  4.418520E-01
          8 1.455600E-02  1.560000E-01
P 1
          1 3.214100E-02  1.000000E+00
HYDROGEN
S 8
          1 4.020000E+02  2.790000E-04
          2 6.024000E+01  2.165000E-03
          3 1.373000E+01  1.120100E-02
          4 3.905000E+00  4.487800E-02
          5 1.283000E+00  1.422990E-01
          6 4.655000E-01  3.309790E-01
          7 1.811000E-01  4.362690E-01
          8 7.279000E-02  1.764400E-01
```

D. LiH, SABS/V5Z-10

```
LITHIUM
S 14
             1  2.95E+04   1.80E-05
             2  4.42E+03   1.41E-04
             3  1.01E+03   7.39E-04
             4  2.85E+02   3.11E-03
             5  9.29E+01   1.11E-02
             6  3.35E+01   3.47E-02
             7  1.30E+01   9.22E-02
             8  5.36E+00   2.00E-01
             9  2.28E+00   3.29E-01
            10  9.94E-01   3.46E-01
            11  4.33E-01   1.43E-01
            12  9.56E-02   5.32E-03
            13  4.47E-02  -2.10E-03
            14  2.06E-02   8.15E-04
S 14
             1  2.95E+04  -3.00E-06
             2  4.42E+03  -2.20E-05
             3  1.01E+03  -1.15E-04
             4  2.85E+02  -4.87E-04
             5  9.29E+01  -1.75E-03
             6  3.35E+01  -5.52E-03
             7  1.30E+01  -1.49E-02
             8  5.36E+00  -3.42E-02
             9  2.28E+00  -6.22E-02
            10  9.94E-01  -9.59E-02
            11  4.33E-01  -1.04E-01
            12  9.56E-02   3.07E-01
            13  4.47E-02   5.79E-01
            14  2.06E-02   2.23E-01
P 1
             1  7.27E-02   1.00E+00
P 8
             1  1.97E+01   5.40E-04
             2  4.62E+00   3.87E-03
             3  1.41E+00   1.52E-02
             4  4.74E-01   4.92E-02
             5  1.76E-01   1.55E-01
             6  7.27E-02   3.60E-01
             7  3.21E-02   4.42E-01
             8  1.46E-02   1.56E-01
P 1
             1  3.21E-02   1.00E+00
HYDROGEN
S 8
             1  4.02E+02   2.79E-04
             2  6.02E+01   2.17E-03
             3  1.37E+01   1.12E-02
             4  3.91E+00   4.49E-02
             5  1.28E+00   1.42E-01
             6  4.66E-01   3.31E-01
             7  1.81E-01   4.36E-01
             8  7.28E-02   1.76E-01
P 1
             1  2.46E-01   1.00E+00
```

E. H_2O, SABS/V5Z-10

```
OXYGEN
S 14
         1  1.642000E+05  2.600000E-05
         2  2.459000E+04  2.050000E-04
         3  5.592000E+03  1.076000E-03
         4  1.582000E+03  4.522000E-03
         5  5.161000E+02  1.610800E-02
         6  1.872000E+02  4.908500E-02
         7  7.393000E+01  1.248570E-01
         8  3.122000E+01  2.516860E-01
         9  1.381000E+01  3.624200E-01
        10  6.256000E+00  2.790510E-01
        11  2.776000E+00  6.355200E-02
        12  1.138000E+00  1.063000E-03
        13  4.600000E-01  1.144000E-03
        14  1.829000E-01  -4.000000E-05
S 14
         1  1.642000E+05  -6.000000E-06
         2  2.459000E+04  -4.600000E-05
         3  5.592000E+03  -2.440000E-04
         4  1.582000E+03  -1.031000E-03
         5  5.161000E+02  -3.688000E-03
         6  1.872000E+02  -1.151400E-02
         7  7.393000E+01  -3.043500E-02
         8  3.122000E+01  -6.814700E-02
         9  1.381000E+01  -1.203680E-01
        10  6.256000E+00  -1.482600E-01
        11  2.776000E+00  9.905000E-03
        12  1.138000E+00  3.842860E-01
        13  4.600000E-01  5.368050E-01
        14  1.829000E-01  2.026870E-01
S 1
         1  1.829000E-01  1.000000E+00
P 8
         1  1.955000E+02  9.180000E-04
         2  4.616000E+01  7.388000E-03
         3  1.458000E+01  3.495800E-02
         4  5.296000E+00  1.154310E-01
         5  2.094000E+00  2.568030E-01
         6  8.471000E-01  3.739380E-01
         7  3.368000E-01  3.434470E-01
         8  1.285000E-01  1.297060E-01
P 1
         1  1.285000E-01  1.000000E+00
HYDROGEN
S 8
         1  4.020000E+02  2.790000E-04
         2  6.024000E+01  2.165000E-03
         3  1.373000E+01  1.120100E-02
         4  3.905000E+00  4.487800E-02
         5  1.283000E+00  1.422990E-01
         6  4.655000E-01  3.309790E-01
         7  1.811000E-01  4.362690E-01
         8  7.279000E-02  1.764400E-01
S 1
         1  1.811000E-01  1.000000E+00
```

F. N_2, SABS/V5Z-6

NITROGEN
S 14

1	1.292000E+05	2.500000E-05
2	1.935000E+04	1.970000E-04
3	4.404000E+03	1.032000E-03
4	1.248000E+03	4.325000E-03
5	4.080000E+02	1.538000E-02
6	1.482000E+02	4.686700E-02
7	5.850000E+01	1.201160E-01
8	2.459000E+01	2.456950E-01
9	1.081000E+01	3.613790E-01
10	4.882000E+00	2.872830E-01
11	2.195000E+00	7.017100E-02
12	8.715000E-01	1.831000E-03
13	3.504000E-01	8.350000E-04
14	1.397000E-01	-6.000000E-06

S 14

1	1.292000E+05	-6.000000E-06
2	1.935000E+04	-4.300000E-05
3	4.404000E+03	-2.270000E-04
4	1.248000E+03	-9.580000E-04
5	4.080000E+02	-3.416000E-03
6	1.482000E+02	-1.066700E-02
7	5.850000E+01	-2.827900E-02
8	2.459000E+01	-6.402000E-02
9	1.081000E+01	-1.139320E-01
10	4.882000E+00	-1.469950E-01
11	2.195000E+00	-7.251000E-03
12	8.715000E-01	3.661830E-01
13	3.504000E-01	5.479080E-01
14	1.397000E-01	2.166450E-01

P 8

1	1.470000E+02	8.920000E-04
2	3.476000E+01	7.082000E-03
3	1.100000E+01	3.281600E-02
4	3.995000E+00	1.082090E-01
5	1.587000E+00	2.480940E-01
6	6.533000E-01	3.745130E-01
7	2.686000E-01	3.484140E-01
8	1.067000E-01	1.283400E-01

IV. ADAPT-VQE ENERGY CONVERGENCE

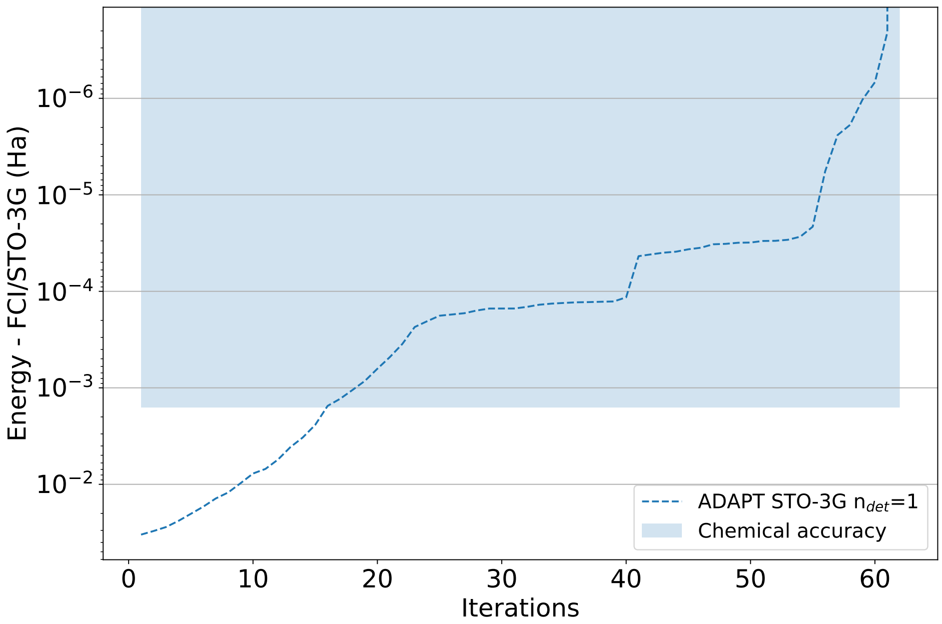

FIG. 1: Error in the ADAPT-VQE energy with respect to the FCI energy with the number of VQE iterations for the H_2O molecule with the STO-3G basis set. The starting point is the HF determinant. The number of required qubits is 12.

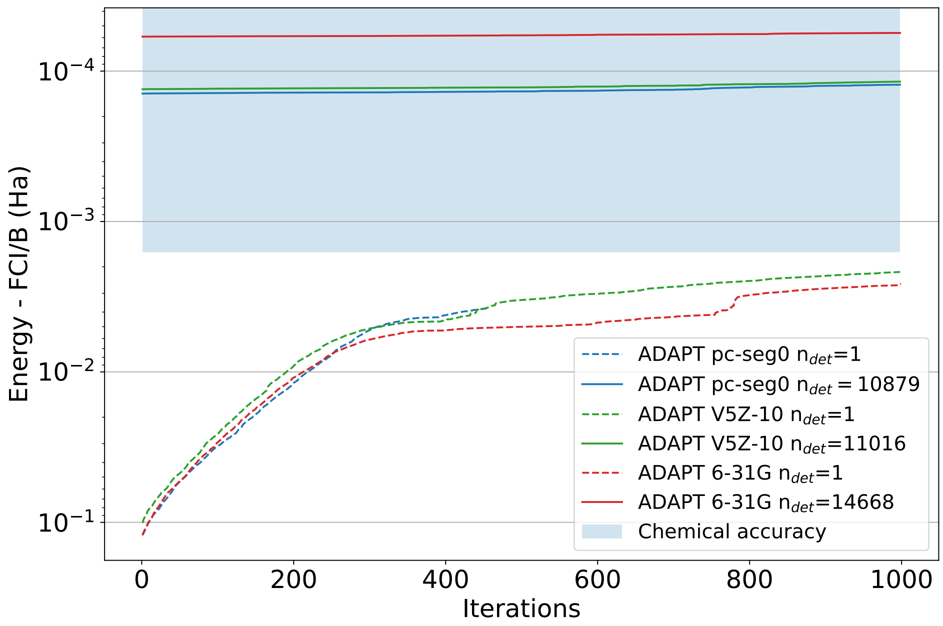

FIG. 2: Error in the ADAPT-VQE energy with respect to the FCI energy with the number of VQE iterations for the H_2O molecule with the pcseg-0, SABS/V5Z-10, and 6-31G basis sets. The quantity $n_{\rm det}$ corresponds to the the number of determinants used for the initial state. For $n_{\rm det} = 1$, the initial state is the HF determinant. For the other cases, we use the CIPSI method to select the determinants. The number of required qubits is 24.

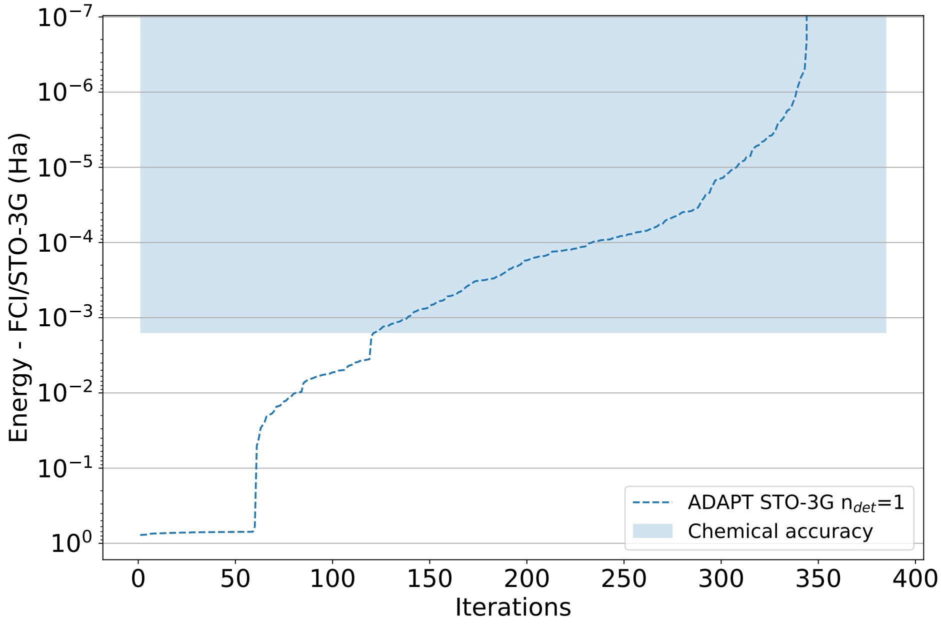

FIG. 3: Error in the ADAPT-VQE energy with respect to the FCI energy with the number of VQE iterations for the N_2 molecule with the STO-3G basis set. The starting point is the HF determinant. The number of required qubits is 16.

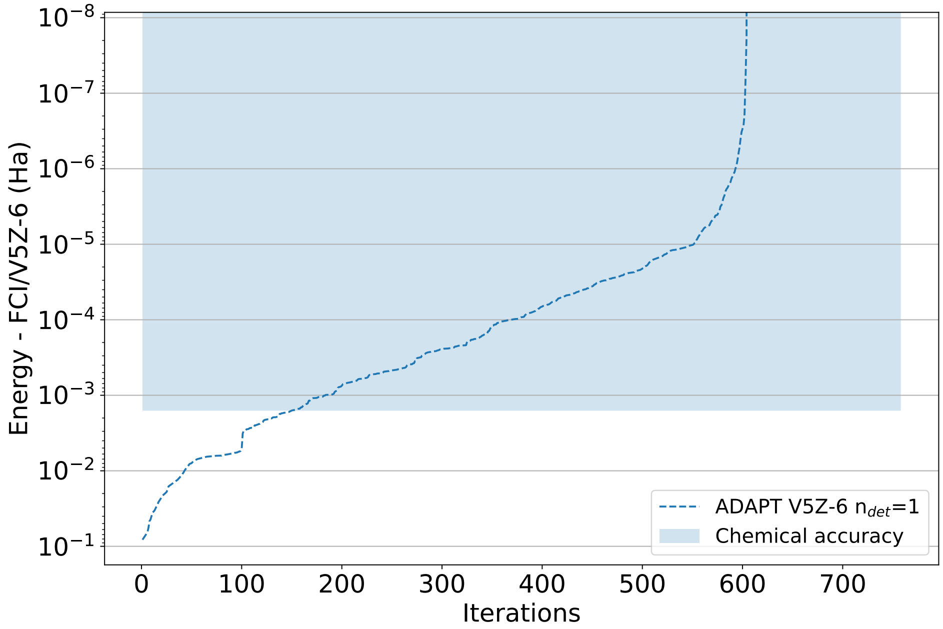

FIG. 4: Error in the ADAPT-VQE energy with respect to the FCI energy with the number of VQE iterations for the N_2 molecule with the SABS/V5Z-6 basis. The starting point is the HF determinant. The number of required qubits is 16.

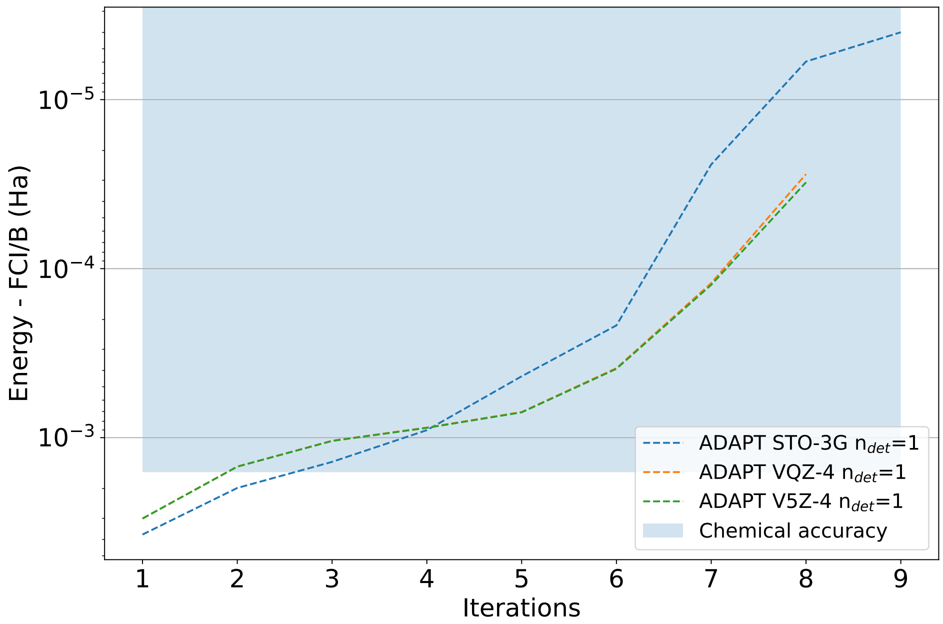

FIG. 5: Error in the ADAPT-VQE energy with respect to the FCI energy with the number of VQE iterations for the LiH molecule with the STO-3G, SABS/VQZ-4, and SABS/V5Z-4 basis sets. The starting point is the HF determinant. The number of required qubits is 10.

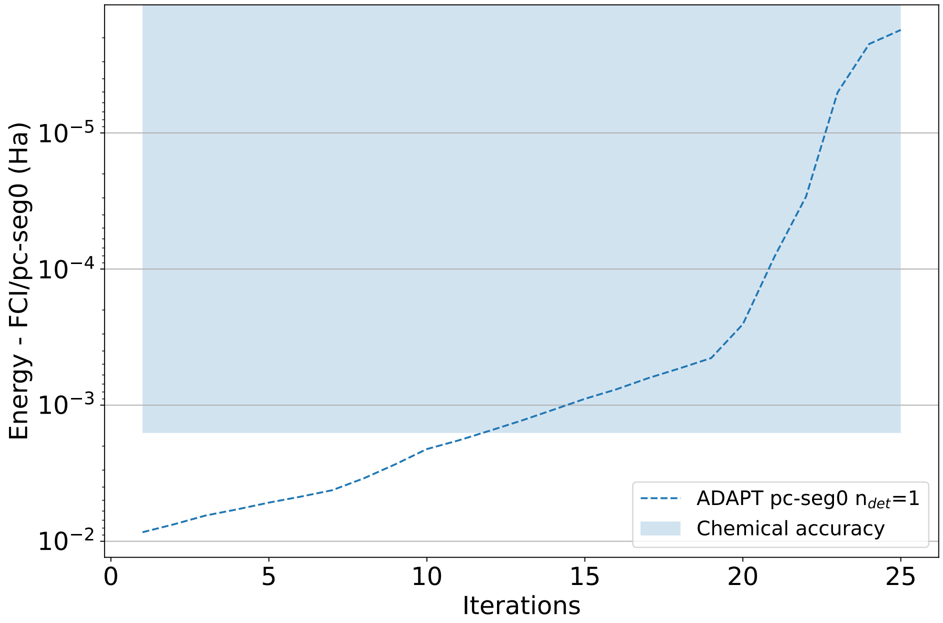

FIG. 6: Error in the ADAPT-VQE energy with respect to the FCI energy with the number of VQE iterations for the LiH molecule with the pcseg-0 basis. The starting point is the HF determinant. The number of required qubits is 14.

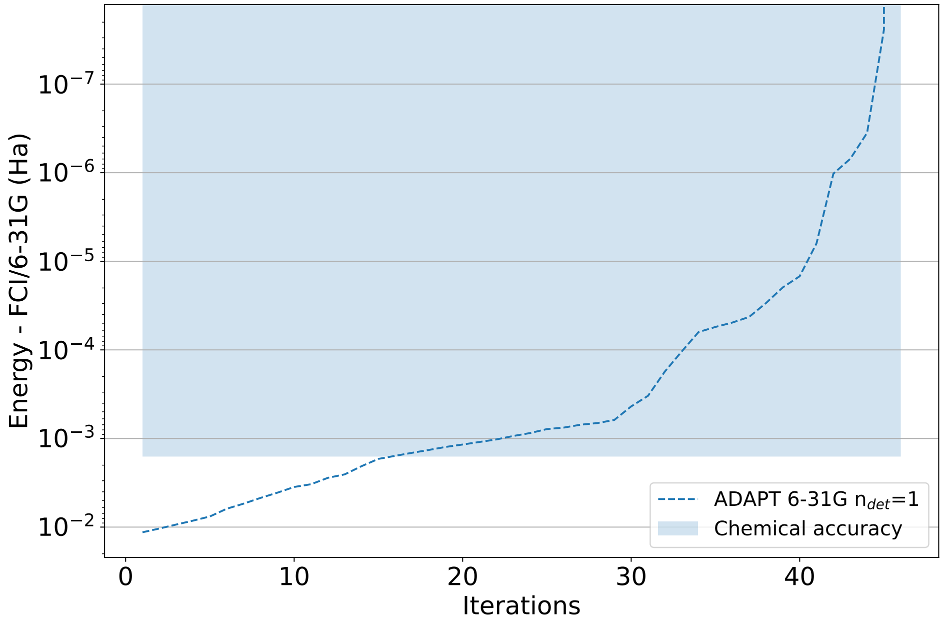

FIG. 7: Error in the ADAPT-VQE energy with respect to the FCI energy with the number of VQE iterations for the LiH molecule with the 6-31G basis set. The starting point is the HF determinant. The number of required qubits is 20.

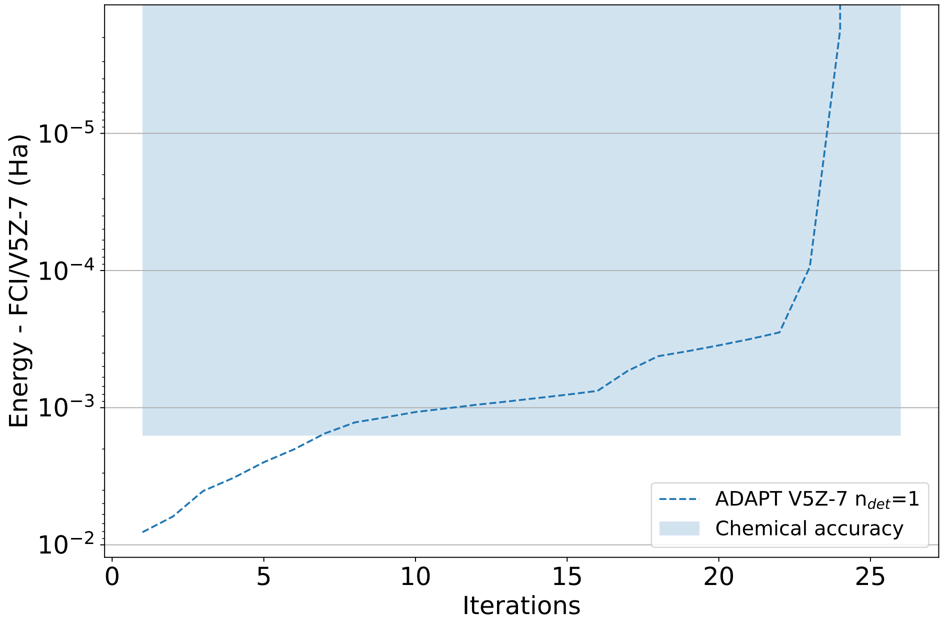

FIG. 8: Error in the ADAPT-VQE energy with respect to the FCI energy with the number of VQE iterations for the LiH molecule with the SABS/V5Z-7 basis. The starting point is the HF determinant. The number of required qubits is 16.

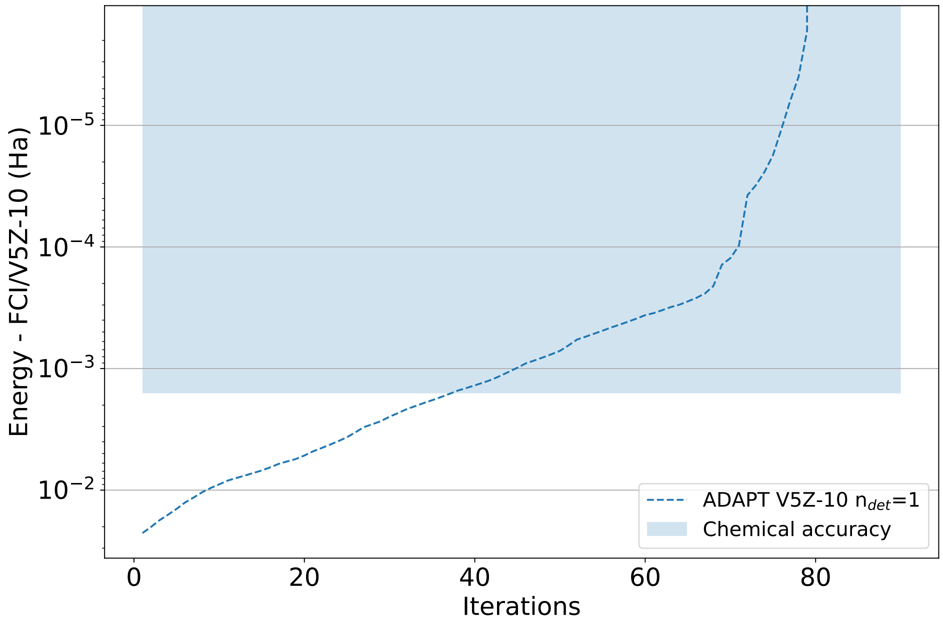

FIG. 9: Error in the ADAPT-VQE energy with respect to the FCI energy with the number of VQE iterations for the LiH molecule with the SABS/V5Z-10 basis set. The starting point is the HF determinant. The number of required qubits is 28.

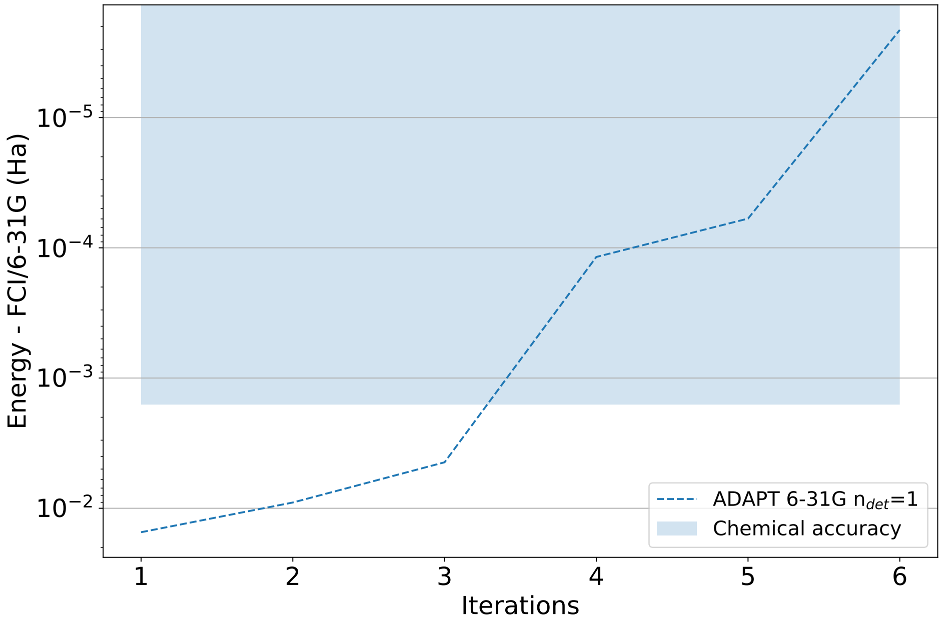

FIG. 10: Error in the ADAPT-VQE energy with respect to the FCI energy with the number of VQE iterations for the H_2 molecule with the 6-31G basis set. The starting point is the HF determinant. The number of required qubits is 8.

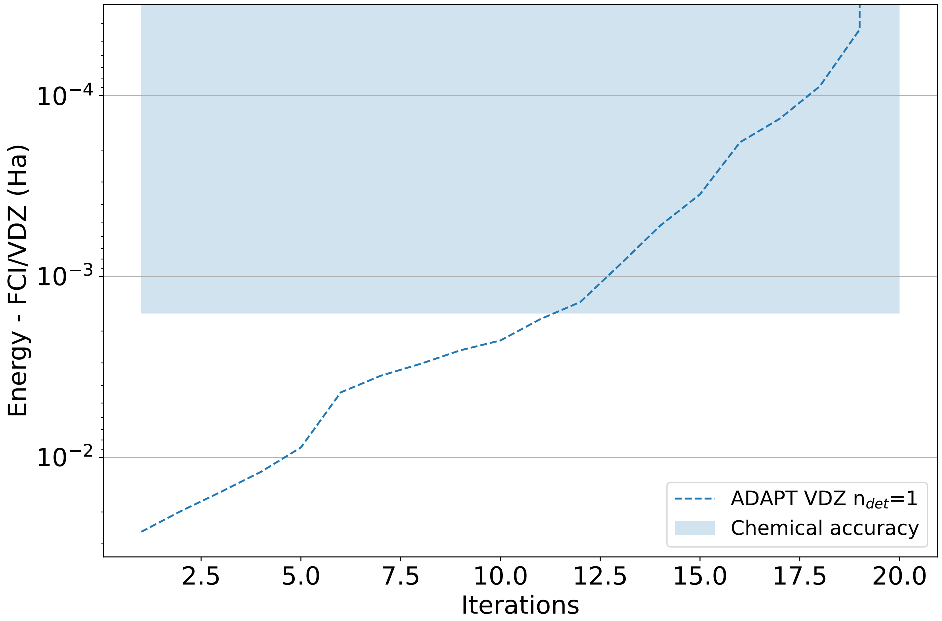

FIG. 11: Error in the ADAPT-VQE energy with respect to the FCI energy with the number of VQE iterations for the H_2 molecule with the cc-pVDZ basis set. The starting point is the HF determinant. The number of required qubits is 20.

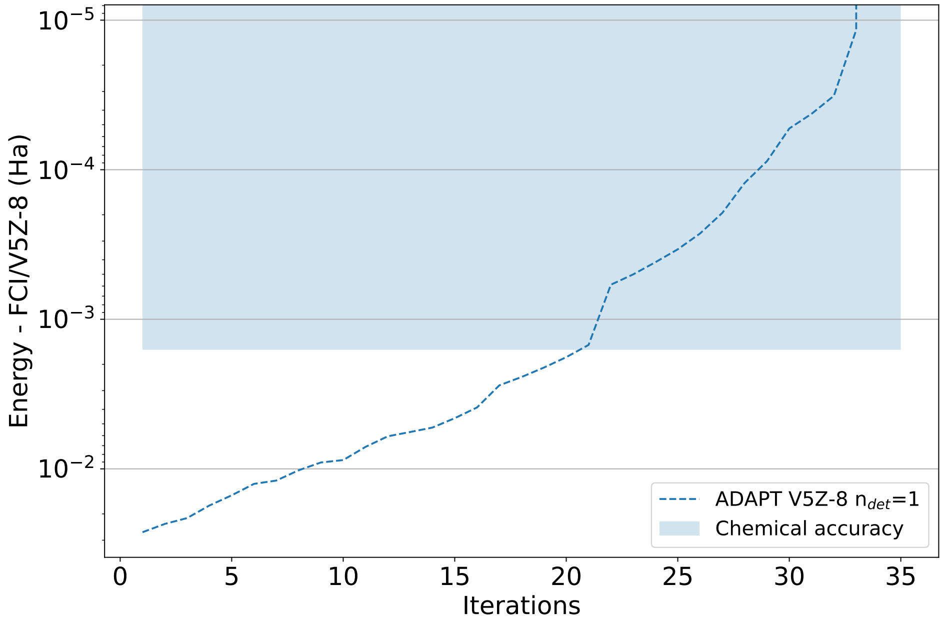

FIG. 12: Error in the ADAPT-VQE energy with respect to the FCI energy with the number of VQE iterations for the H_2 molecule with the SABS/V5Z-8. The starting point is the HF determinant. The number of required qubits is 24.

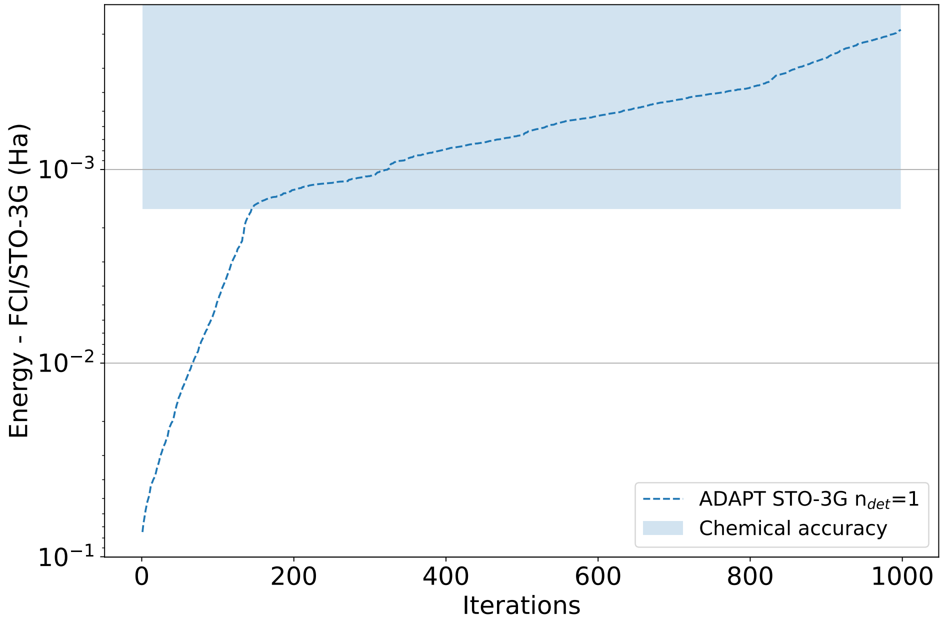

FIG. 13: Error in the ADAPT-VQE energy with respect to the FCI energy with the number of VQE iterations for the H_8 molecule with the STO-3G basis set. The starting point is the HF determinant. The number of required qubits is 16.

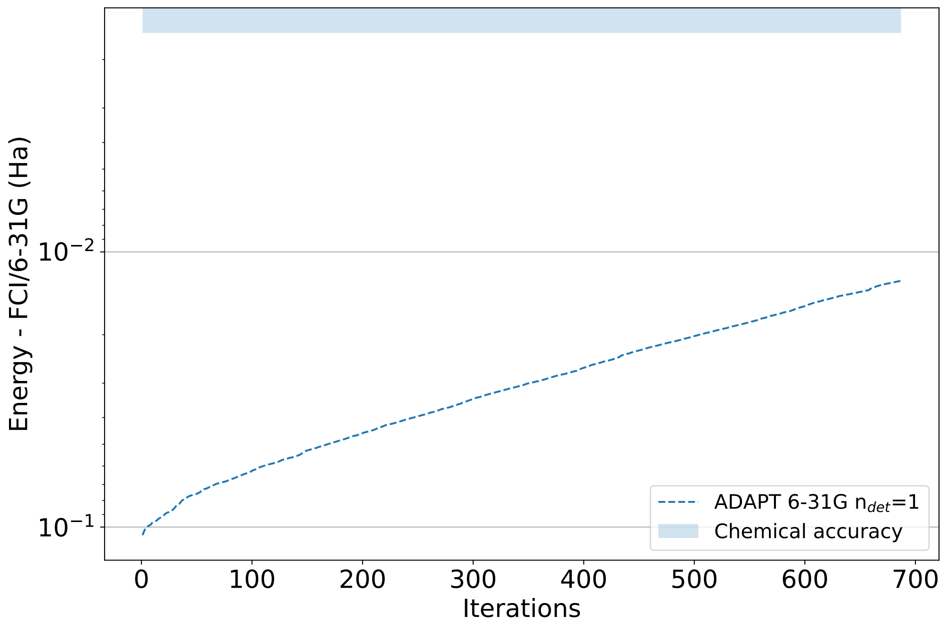

FIG. 14: Error in the ADAPT-VQE energy with respect to the FCI energy with the number of VQE iterations for the H_8 molecule with the 6-31G basis-set. The starting point is the HF determinant. The number of required qubits is 32.

[1] P. Jordan and E. P. Wigner, "Über das Paulische äquivalenzverbot," in *The Collected Works of Eugene Paul Wigner*, pp. 109–129, Springer, 1993.
[2] C. D. Batista and G. Ortiz, "Generalized Jordan-Wigner transformations," *Physical Review Letters*, vol. 86, no. 6, p. 1082, 2001.
[3] A. Peruzzo, J. McClean, P. Shadbolt, M.-H. Yung, X.-Q. Zhou, P. J. Love, A. Aspuru-Guzik, and J. L. O'brien, "A variational eigenvalue solver on a photonic quantum processor," *Nature Communications*, vol. 5, no. 1, pp. 1–7, 2014.
[4] A. Y. Kitaev, "Quantum measurements and the abelian stabilizer problem," *Electron. Colloquium Comput. Complex.*, vol. TR96, 1995.
[5] M. A. Nielsen and I. L. Chuang, *Quantum computation and quantum information.* Cambridge University Press, 2010.
[6] A. Aspuru-Guzik, A. D. Dutoi, P. J. Love, and M. Head-Gordon, "Simulated quantum computation of molecular energies," *Science*, vol. 309, no. 5741, pp. 1704–1707, 2005.
[7] J. D. Whitfield, J. Biamonte, and A. Aspuru-Guzik, "Simulation of electronic structure hamiltonians using quantum computers," *Molecular Physics*, vol. 109, no. 5, pp. 735–750, 2011.
[8] J. Tilly, H. Chen, S. Cao, D. Picozzi, K. Setia, Y. Li, E. Grant, L. Wossnig, I. Rungger, G. H. Booth, *et al.*, "The variational quantum eigensolver: a review of methods and best practices," *Physics Reports*, vol. 986, pp. 1–128, 2022.
[9] H. R. Grimsley, S. E. Economou, E. Barnes, and N. J. Mayhall, "An adaptive variational algorithm for exact molecular simulations on a quantum computer," *Nature Communications*, vol. 10, p. 3007, 2019.
[10] C. Feniou, M. Hassan, D. Traoré, E. Giner, Y. Maday, and J.-P. Piquemal, "Overlap-ADAPT-VQE: practical quantum chemistry on quantum computers via overlap-guided compact ansätze," *Communications Physics*, vol. 6, p. 192, jul 2023.
[11] Y. S. Yordanov, V. Armaos, C. H. Barnes, and D. R. Arvidsson-Shukur, "Qubit-excitation-based adaptive variational quantum eigensolver," *Communications Physics*, vol. 4, no. 1, p. 228, 2021.
[12] C. Feniou, M. Hassan, D. Traore, E. Giner, Y. Maday, and J.-P. Piquemal, "Overlap-ADAPT-VQE: Practical Quantum Chemistry on Quantum Computers via Overlap-Guided Compact Ansätze," *Communications Physics*, vol. 6, 2023.
[13] E. Giner, D. Traore, B. Pradines, and J. Toulouse, "Self-consistent density-based basis-set correction: How much do we lower total energies and improve dipole moments?," *Journal of Chemical Physics*, vol. 155, no. 4, p. 044109, 2021.
